# The Relationship Between Driving Performance and Lower Limb Motor Function After Total Knee Arthroplasty Using a Driving Simulator: A Pilot Study on Elucidating Factors Influencing Accelerator and Brake Operations

**DOI:** 10.3390/life15050768

**Published:** 2025-05-11

**Authors:** Kazuya Okazawa, Satoshi Hamai, Tsutomu Fujita, Yuki Nasu, Shinya Kawahara, Yasuharu Nakashima, Hitoshi Ishikawa, Hiromi Fujii, Hiroshi Katoh

**Affiliations:** 1 Department of Rehabilitation, Kyushu University Hospital, Fukuoka 812-8582, Japan; 2 Graduate School of Health Sciences, Yamagata Prefectural University of Health Sciences, Yamagata 990-2212, Japanhikato@yachts.ac.jp (H.K.); 3 Department of Orthopedic Surgery, Graduate School of Medical Sciences, Kyushu University, Fukuoka 812-8582, Japan

**Keywords:** total knee arthroplasty, driving simulator, gait

## Abstract

Background: The aging population in Japan has led to an increase in traffic accidents involving elderly drivers, highlighting the need for measures to enhance driving safety. Post-total knee arthroplasty (TKA) patients must regain their driving ability to maintain independence; however, clear guidelines for driving resumption are lacking. This study assessed the movement time (MT) and brake pedal force (BPF) using a driving simulator and investigated their associations with lower limb motor function. Methods: This single-center prospective cohort study included 21 patients (mean age: 66.7 ± 7.4 years) who underwent right TKA and intended to resume driving. Driving ability was assessed on postoperative day 13 using a driving simulator to measure MT and BPF. Physical function was evaluated using the following parameters: range of motion (ROM), muscle strength, gait parameters, and pain assessment. Pearson’s correlation and multiple regression analyses were performed to identify significant associations. Results: MT was significantly correlated with knee extension strength (r = −0.56, *p* = 0.02) and walking ratio (r = 0.55, *p* = 0.03). BPF was significantly correlated with walking ratio (r = 0.52, *p* = 0.04) and pain levels VAS (r = −0.54, *p* = 0.02). Multiple regression analysis identified walking ratio (β = 0.54, *p* = 0.02) as a significant predictor of MT. For BPF, significant predictors included walking ratio (β = 0.49, *p* = 0.03) and VAS (β = −0.54, *p* = 0.02). Discussion: The findings of this study suggest that MT is associated with walking ratio, while BPF is significantly associated with both walking ratio and VAS scores. In particular, walking ratio was found to have a significant impact on both MT and BPF, indicating that it may be an important factor influencing postoperative driving performance. Conclusion: Improvement in the walking ratio and pain management affect accelerator and brake operation during driving after TKA.

## 1. Introduction

In Japan, the rapid aging of the population increased the number of serious traffic accidents involving elderly drivers [1]. Consequently, specific measures are required to reduce the risk of accidents among elderly drivers. One such measure is the ability to apply an emergency brake appropriately in critical situations [2,3]. However, previous studies have reported that in older adults, muscle weakness may delay the brake reaction time (BRT), which refers to the time from recognizing a hazard to activating the brake [4,5]. For elderly patients undergoing total knee arthroplasty (TKA), resuming driving is important for maintaining independence in daily life and returning to work [6]. However, guidelines, such as those provided by the Osteoarthritis Research Society International, do not establish clear criteria regarding the timing of driving resumption after TKA [7,8].

Previous studies that measured BRT using driving simulators have reported that the average time to driving resumption after TKA was approximately 4.5 weeks [9]. However, these studies used different types of driving simulators, resulting in considerable variations in BRT measurements (ranging from 430 to 1330 ms), making standardization challenging [3,9,10]. To address this issue, other studies have examined additional driving performance parameters, such as the movement time (MT) and brake pedal force (BPF) [11,12,13]. MT is defined as the time taken for a driver to switch from the accelerator to the brake; it does not include the neural reaction time, which refers to the time from hazard recognition to the initiation of the braking motion. The BPF represents the force applied to the brake pedal, with greater force corresponding to stronger braking effectiveness. The BPF is a critical factor in performing emergency braking maneuvers [3]. Therefore, the MT and BPF are lower-limb motor function parameters that do not include the neural reaction time. However, studies on the MT [14,15] and BPF [3,16] in patients with TKA are limited. Furthermore, most previous studies using driving simulators have focused on the identification of the recovery timeline of each driving function postoperatively rather than the examination of their relationship with lower-limb motor function.

Therefore, this study evaluated MT and BPF using a driving simulator in post-TKA patients and investigated their associations with physical function. We hypothesized that postoperative physical functions, including range of motion of the knee joint, muscle strength, gait ability, and pain, would significantly influence pedal operation during simulated driving.

## 2. Methods

### 2.1. Study Design

This study was conducted as a single-center prospective cohort study.

### 2.2. Participants

This study included 21 individuals (11 men and 10 women; mean age: 66.7 ± 7.4 years; mean body mass index: 28.5 ± 4.3 kg/m^2^) who underwent right TKA at our institution between January 2023 and May 2024 and expressed a desire to resume driving postoperatively (Table 1).

**Figure 1:**
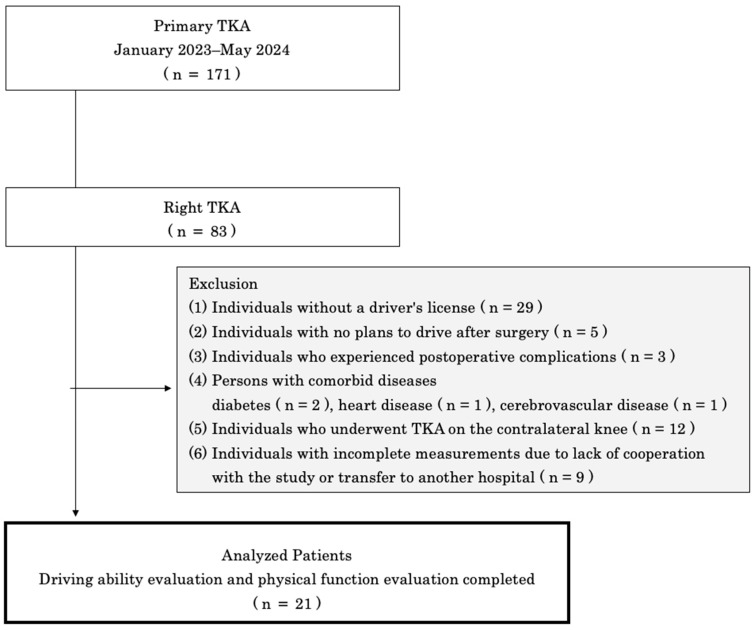
Selection criteria for the participants (flowchart).

The exclusion criteria were as follows: (1) individuals without a valid driver’s license; (2) those who did not plan to resume driving postoperatively; (3) those who experienced postoperative complications; (4) those with comorbid conditions, such as diabetes mellitus, heart disease, and cerebrovascular disease; (5) those who had undergone contralateral TKA; and (6) those who were unable to participate in the study or were transferred to another hospital, resulting in incomplete data collection (Figure 1).

Rehabilitation during hospitalization was performed using our institution’s clinical pathway to ensure standardized treatment. This study was approved by the Ethics Committee of our institution (22104-00). Furthermore, all participants received a detailed explanation of the study’s significance and objectives and provided informed consent before participation.

### 2.3. Tasks

The task involved assessing driving ability using a driving simulator.

### 2.4. Measurement Procedures

The outcome measures included the MT and BPF for driving ability assessment and range of motion (ROM) of the knee joint, lower-limb muscle strength, gait ability, and pain for physical function assessment. All assessments were performed on postoperative day 13 following TKA. Regarding the assessment timing, the average length of hospital stay at our institution is 13.9 ± 1.3 days. Furthermore, previous studies have reported that patients can resume driving 2 weeks after TKA [6]. Therefore, the assessment in this study was conducted on postoperative day 13.

#### 2.4.1. MT and BPF

**Figure 2:**
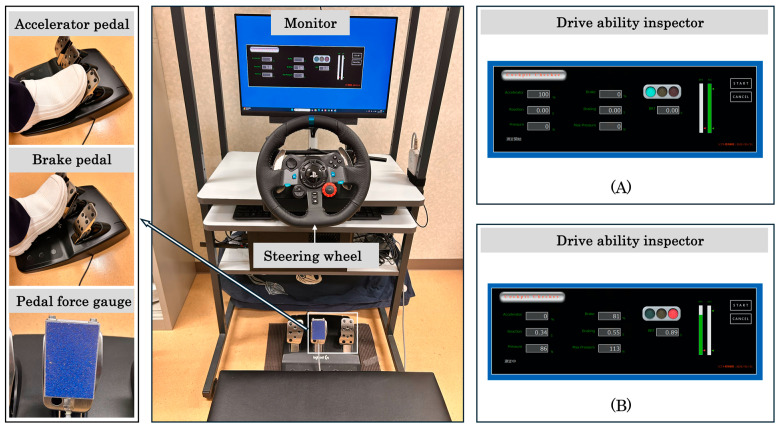
Driving simulator. The driving simulator comprises a monitor, steering wheel, accelerator pedal, brake pedal, a force sensor embedded in the brake pedal, and the Drive Ability Inspector software for driving ability assessment. When the accelerator pedal is pressed beyond 90% of its range, a green light illuminates (**A**). After a predetermined period, the software randomly determines the delay time, after which a red light illuminates (**B**).

The MT and BPF were measured using a driving simulator (Honda Motor, Tokyo, Japan), which has been widely used in recent rehabilitation studies [17,18], and the Drive Ability Inspector software Ver.1.0. (Manage Business, Tokyo, Japan) for the driving ability assessment (Figure 2).

**Figure 3:**
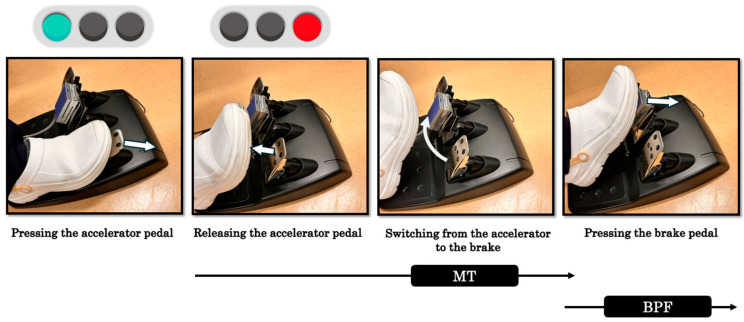
MT and BPF. MT (movement time): The time required from foot-off from the accelerator pedal to the point at which the brake pedal displacement exceeds 10% of its maximum range. Both pedals had a resolution of 15 bits and a stroke length of approximately 2 cm. The absolute measurement error of the MT during pedal operation is within ±0.01 s. BPF (brake pedal force): The maximum force applied to the brake pedal. The pedal force sensor used in this study has a maximum capacity of 1500 N, with an absolute measurement error of within ±1% of full scale.

A 27-inch monitor was positioned in front of the steering wheel to simulate the driving environment. The monitor was adjusted to match the participant’s eye level, and the steering wheel was positioned to maintain a natural driving posture. To measure the BPF, a PK2-1500N pedal force sensor (Imada, Tokyo, Japan) was installed on the surface of the brake pedal. For the task, the participants were first instructed to place their foot on the accelerator pedal at their preferred timing. Next, they were required to depress the accelerator pedal until the signal turned green, and after the signal turned red, they were to quickly switch to the brake pedal and apply maximum force for an emergency stop. In this task, the MT (seconds) was defined as the time from foot-off from the accelerator pedal to the point at which the brake pedal displacement exceeded 10% of its maximum range [19]. The BPF (N) was defined as the maximum force applied to the brake pedal [3,20] (Figure 3). The participants performed three practice trials before completing 10 consecutive test trials, as previously described [21,22]. The interval between trials was set at 5 s, and the average of the 10 trials was used for analysis.

#### 2.4.2. ROM and Lower-Limb Muscle Strength

ROM was assessed using a goniometer (SPR-625R, SAKAI Medical, Tokyo, Japan) to measure knee joint flexion and extension ROM in 5° increments. The testing position was set at 90° hip flexion and 60° knee flexion. The reliability of measurements using the goniometer has been reported to have a measurement error of within ±0.39° [23]. Lower-limb muscle strength was evaluated using an isokinetic dynamometer (COMBIT CB-2, Minato Medical Science, Osaka, Japan). The reliability of this device has been reported to have a measurement error from 5.1% to 9.3% [24]. The measurement posture was set at 90° hip flexion and 60° knee flexion. The participants performed a 3 s maximum isometric contraction, and the highest torque value was recorded. The measured values were normalized to the body weight (Nm/kg) [25].

#### 2.4.3. Gait Performance

A mat-based foot pressure measurement system (WalkWay MW-1000, Anima, Tokyo, Japan) was used to assess gait performance [26,27,28]. This device’s sensor resolution is 1 cm^2^, and the measurement load ranges from 0.2 to 8.0 kg/cm^2^. The test was performed under natural walking conditions, with four trials. The measured parameters included gait speed (cm/s), stride length (cm), and gait ratio (cm/steps/min). The WalkWay MW-1000 (60 cm in width and 7.2 m in length) was installed at the center of a 10 m walkway, as previously described [29]. The average values of each parameter were calculated based on the foot pressure data obtained from the four trials.

#### 2.4.4. Visual Analog Scale (VAS) and Tampa Scale of Kinesiophobia (TSK)

Subjective pain during movement was assessed using the VAS. Kinesiophobia, or fear of movement related to pain, was evaluated using the TSK. The TSK score ranged from 17 to 68, with higher scores indicating a greater degree of fear-avoidance behavior [30].

#### 2.4.5. Statistical Analyses

Data normality was assessed using the Shapiro–Wilk test. The correlation between MT, BPF, and each physical function was examined using Pearson’s correlation coefficient (r). Furthermore, multiple regression analysis was performed to investigate the relationships among MT, BPF, and physical function. Multicollinearity between variables was assessed using the variance inflation factor (VIF). To assess the adequacy of the sample size, post hoc power analysis was performed using G*Power (version 3.1.9.7) and Cohen’s method [31]. Statistical analyses were performed using JMP (version 18; JMP Statistical Discovery, Cary, NC, USA). The significance level was set at 5%.

## 3. Results

### 3.1. MT, BPF, and Physical Function

The MT and BPF were 0.35 ± 0.11 s and 137.53 ± 34.02 N, respectively. Regarding physical function, the knee extension strength, walking ratio, and VAS were 0.27 ± 0.13 Nm/kg, 0.48 ± 0.11 cm/steps/min, and 31.53 ± 24.07, respectively (Table 2).

### 3.2. Correlation Among MT, BPF, and Physical Function

MT showed a significant correlation with knee extension strength (r = −0.56, *p* = 0.02) and walking ratio (r = 0.55, *p* = 0.03). BPF showed a significant correlation with walking ratio (r = 0.52, *p* = 0.04) and VAS (r = −0.54, *p* = 0.02) (Table 3).

### 3.3. Multiple Regression Analysis of MT, BPF, and Physical Function

MT exhibited a significant correlation with knee extension strength and walking ratio, whereas BPF exhibited a significant correlation with the walking ratio and VAS. Therefore, multiple regression analysis was performed with the inclusion of these parameters. The variables significantly associated with MT were the walking ratio (β = 0.54, *p* = 0.02). The variables significantly associated with BPF were walking ratio (β = 0.49, *p* = 0.03) and VAS (β = −0.54, *p* = 0.02) (Table 4).

### 3.4. Validity of Sample Size

The power analysis for the multiple regression model of MT (R^2^ = 0.44, α = 0.05, sample size = 21) yielded a power (1-β) of 0.71. For the multiple regression model of BPF (R^2^ = 0.44, α = 0.05, total sample size = 21), the power (1-β) was 0.71.

## 4. Discussion

In previous studies using a driving simulator after TKA, the relationship between lower-limb physical function and MT and BPF recovery was not clarified. Therefore, this study examined the relationship between MT, BPF, and lower-limb physical function. The results revealed that MT was significantly associated with knee extension strength and the walking ratio, whereas BPF was significantly associated with the walking ratio and VAS.

### 4.1. Clinical Characteristics of MT, BPF, and Physical Function

According to previous studies [10], MT 2–4 weeks after TKA has been reported to range from 0.29 to 0.36 s. The results of this study fell within this range. Furthermore, Nizam et al. reported that resumption of driving is possible 2 weeks after TKA [32]. Therefore, the results of this study suggest that by postoperative day 13, MT had recovered to a level sufficient for driving resumption. BPF exhibited lower values than those reported in previous systematic reviews. However, the measurement periods in these reports ranged from 8 weeks to 1 year after surgery, which likely accounts for the observed differences [9]. Furthermore, since 2011, the European Union has mandated the installation of brake assist units in all newly registered vehicles [9]. This technology ensures that the BPF is optimized, even when insufficient force is applied. Considering this technical background, evaluating postsurgical BPF could serve as an essential indicator for ensuring vehicle safety [3].

The knee ROM in this study was relatively favorable compared with the extension values of −6° to −9° and flexion values of 93–111° reported in previous studies 10 days to 1 month after surgery [33,34,35]. In contrast, the knee extension strength was lower than the 0.66 Nm/kg value reported 3 weeks after surgery [36]. This difference is likely attributable to variations in the measurement timing, similar to BPF. Similarly, it has been reported that knee extension strength decreases to approximately 62% of preoperative values by day 10 post-TKA [36]. In particular, quadriceps strength is affected by muscle atrophy and impaired neuromuscular control due to surgical invasion, with early postoperative strength loss being pronounced [37,38].

Regarding walking ability, the walking ratio was lower than the 0.52–0.61 cm/steps/min range reported for healthy elderly individuals of the same age group in previous studies [39,40]. The walking ratio, which indicates the coordination of stride length and walking rhythm [41], is generally associated with aging, muscle weakness, pain, and decreased balance ability [39]. Therefore, muscle weakness and pain due to surgical trauma decreased the walking ratio observed in this study.

Regarding pain, the VAS score was comparable to the 37.0 ± 5.0 value reported in previous studies 2 weeks after TKA [42]. However, patients experiencing severe pain after TKA may exhibit kinesiophobia and avoidance behaviors, which may delay rehabilitation progress [43]. The cutoff score for assessing the impact of kinesiophobia on rehabilitation is considered to be 37 points on the TSK [37], indicating that the influence of kinesiophobia on rehabilitation in this study was minimal.

### 4.2. Relationships Between MT, BPF, and Physical Function

MT exhibited a negative correlation with knee extension strength and a positive correlation with walking ratio. This suggests that a decrease in knee extension strength and an increase in the walking ratio are associated with an extension of the MT during braking during driving. Appropriate muscle strength is required for switching quickly from the accelerator to the brake pedal during driving. Therefore, improving knee extension strength is considered essential for shortening the MT. Regarding the walking ratio, approximately 0.6 cm/steps/min is considered the most energy-efficient rate, with increased or decreased values leading to decreased efficiency [44]. In this study, the average walking ratio was 0.48. Among those with a walking ratio < 0.6, those with a walking ratio closer to 0.6 exhibited a tendency for MT to shorten, whereas those with a walking ratio > 0.6 tended to have extended MT.

Furthermore, BPF was positively correlated with the walking ratio and negatively correlated with the VAS. This indicates that an increased walking ratio and a decreased VAS score are associated with an increased BPF during braking operations. Similarly to MT, individuals with a walking ratio closer to 0.6 are likely to exhibit more stable muscle strength during the braking motion. Regarding pain, it has been reported that pain can suppress muscle strength through its effects on the central nervous system [45]. This phenomenon is considered significant for the recovery of BPF during the early postoperative phase.

### 4.3. Effects of Physical Function on MT, BPF

The results of the multiple regression analysis revealed that the significant variable for MT was the walking ratio (β = 0.54, *p* = 0.02). In contrast, the significant variables for BPF were the walking ratio (β = 0.49, *p* = 0.03) and the VAS score (β = −0.54, *p* = 0.02). The VIF values for all variables were below 2.0, indicating no multicollinearity issues, as a VIF value of 5 or higher is typically considered indicative of multicollinearity [39]. Therefore, it can be concluded that there is no collinearity between the walking ratio and VAS score, and these variables independently contribute to MT and BPF. Notably, the walking ratio significantly affected MT and BPF, suggesting its critical role as a factor for postoperative driving ability. Therefore, improving the walking ratio could improve MT and BPF in driving tasks, highlighting its importance as an intervention target in rehabilitation. Furthermore, comparing the magnitude of the β values, it was observed that the VAS score had a higher β value than the walking ratio for BPF, indicating that the improvement of VAS is likely more important. Based on these findings, it can be concluded that for safe driving resumption after surgery, improving the walking ratio and managing pain through rehabilitation are crucial. 

### 4.4. Limitations and Future Directions

This study has several limitations that should be acknowledged. First, the post hoc power analysis revealed that the power (1-β) for the primary outcomes—MT and BPF—was 0.71, which is slightly lower than the recommended power of 0.8 [24]. This suggests that the sample size should be increased to enhance the reliability of this study. Second, although this study evaluated driving ability on postoperative day 13 after TKA, the actual timing of driving resumption and long-term driving ability were not examined. Third, patients with comorbidities or postoperative complications were excluded; thus, the findings of this study may not be generalizable to all patients who underwent TKA. Fourth, the driving simulator does not fully replicate real-world traffic conditions. Therefore, it may not guarantee actual on-road driving performance. Fifth, driving ability may be influenced by physical function, psychological status, overall motor coordination, and past driving experience, among other factors. Future studies should incorporate a more comprehensive assessment that considers these factors.

## 5. Conclusions

Improvement in the walking ratio and pain management affect accelerator and brake operation during driving after TKA.

## Figures and Tables

**Table 1:** Basic demographic characteristics of the participants (n = 21).

Sex (male/female)	11	/	10
Age (years)	66.7	±	7.4
Height (cm)	159.8	±	8.5
Weight (kg)	72.9	±	13.4
BMI (kg/m^2^)	28.5	±	4.3

Values are presented as mean ± SD; BMI: body mass index.

**Table 2:** Evaluation of driving ability and physical function.

Measures			
MT (s)	0.35	±	0.11
BPF (N)	137.53	±	34.02
Knee flexion ROM (°)	116.19	±	12.34
Knee extension ROM (°)	−5.00	±	5.24
Knee flexion muscle strength (Nm/kg)	0.31	±	0.13
Knee extension muscle strength (Nm/kg)	0.27	±	0.13
Walking speed (cm/s)	66.41	±	24.40
Stride length (cm)	86.36	±	20.27
Walking ratio (cm/steps/min)	0.48	±	0.11
VAS	31.53	±	24.07
TSK	34.24	±	8.07

MT: movement time, BPF: brake pedal force, ROM: range of motion, VAS: visual analog scale, TSK: Tampa scale for kinesiophobia.

**Table 3:** Correlation between driving ability and physical function.

	MT			BPF		
	r	95% CI	*p*-Value	r	95% CI	*p*-Value
Knee flexion ROM (°)	−0.40	−0.79, 0.12	0.12	<0.00	−0.14, 0.75	1.00
Knee extension ROM (°)	−0.12	−0.66, 0.37	0.66	0.07	−0.60, 0.40	0.79
Knee flexion muscle strength (Nm/Kg)	−0.40	−0.78, 0.15	0.15	0.01	−0.44, 0.65	0.97
Knee extension muscle strength (Nm/Kg)	−0.56	−0.88, −0.18	0.02 *	−0.12	−0.35, 0.65	0.68
Walking speed (cm/s)	0.29	−0.47, 0.59	0.27	0.19	−0.29, 0.68	0.49
Stride length (cm)	0.20	−0.29, 0.71	0.45	0.20	−0.06, 0.79	0.45
Walking ratio (cm/steps/min)	0.55	0.10, 0.86	0.03 *	0.52	0.03, 0.82	0.04 *
VAS	−0.11	−0.48, 0.58	0.68	−0.54	−0.92, −0.42	0.02 *
TSK	−0.30	−0.80, 0.09	0.27	0.11	−0.54, 0.48	0.68

MT: movement time, BPF: brake pedal force, ROM: range of motion, VAS: visual analog scale, TSK: Tampa scale for kinesiophobia, 95% CI: 95% confidence interval, * *p* < 0.05.

**Table 4:** Results of multiple regression analysis of physical function affecting driving function evaluation (n = 21).

Dependent Variables	Independent Variables	Unstandardized Coefficient	95% CI for B (Lower Limit, Upper Limit)	Standardized Coefficient	95% CI for β (Lower Limit, Upper Limit)	*p*-Value	VIF	R^2^
		B		β				
MT								
	Intercept	0.25	0.14, 0.31		0.02, 0.02			0.44
	Knee extension strength (Nm/Kg)	−0.001	−0.002, −0.0001	−0.42	−0.886, 0.044	0.07	1.00	
	Walking ratio (cm/steps/min)	241.04	32.51, 449.56	0.54	0.077, 1.004	0.02 *	1.00	
BPF								
	Intercept	87.79	19.62, 155.96		−3.78, 0.51			0.44
	Walking ratio (cm/steps/min)	151,747.48	12,271.66, 291,223.3	0.49	0.0398, 0.9456	0.03 *	1.03	
	VAS	−0.70	−1.29, −0.12	−0.54	−0.9972, −0.0922	0.02 *	1.03	

MT: movement time, BPF: brake pedal force, ROM: range of motion, VAS: visual analog scale, B: regression coefficient, β: standardized regression coefficient, R^2^: coefficient of determination, VIF: variance inflation factor, 95% CI: 95% confidence interval, * *p* < 0.05.

## Data Availability

The raw data supporting the conclusions of this article will be made available by the authors upon request.
